# Priority evaluation factors for blockchain application services in public sectors

**DOI:** 10.1371/journal.pone.0279445

**Published:** 2023-03-02

**Authors:** Joongyeup Lee, Beomsoo Kim, Ae Ri Lee

**Affiliations:** 1 Software Policy Research Team, Software Policy & Research Institute, Seongnam, Republic of Korea; 2 Graduation School of Information, Yonsei University, Seoul, Republic of Korea; 3 Department of Business Administration, Sangmyung University, Seoul, Republic of Korea; University of Pisa, ITALY

## Abstract

Blockchain is rapidly becoming established as the core technology of the Fourth Industrial Revolution. By combining blockchain to improve processes in existing industries, innovative new services will emerge, but services not effectively applied by blockchain will also develop. This study investigated the factors to be considered when applying the characteristics of blockchain technology to business. We developed a framework of blockchain service utility evaluation indexes using the analytic hierarchy process method. The Delphi method is used to identify highly effective blockchain application service cases by applying the evaluation framework to actual use cases in the public sector. By proposing a framework of utility evaluation factors for blockchain application services, this study provides a systematic foundation for blockchain business review. We address the question of “why blockchain should be applied to this service” by providing a more comprehensive approach than existing research, such as a fragmentary decision tree. Blockchains are expected to become more active along with the full-scale digital transformation of industries, and thus, we must examine the ways to broadly use blockchain as a base technology in a form applicable to the diverse industries and societies constituting the digital economy. Accordingly, this study presents an evaluation solution for promoting efficient policies and developing successful blockchain application services.

## Introduction

Global blockchain spending will reach around $19 billion by 2024, from $6.6 billion in 2021, and blockchain spending will continue to grow over the 2020–2024 forecast period at a five-year compound annual growth rate of 48.0% [1]. Although the market share is still small when compared with that of other new technologies, such as big data and cloud, the rapid market growth demonstrates the market’s high expectations for blockchain. This expectation is based on the unique characteristics and value of blockchain technology (hereinafter BCT).

Blockchain has the characteristics of decentralization, high security level, and transparency, and it can be used as a core infrastructure technology to develop new growth industries. Various fields now have fascinating insights into the advantages of blockchain, and some papers have discussed the major potential of blockchains in Industry 4.0 [2]. By using BCT, we can induce cost savings during multilateral transactions and ensure convenience and safety in data usage. It can also be key through the Fourth Industrial Revolution by enabling autonomous cooperation support between IoT devices. Based on these characteristics, many countries are continuously discovering cases of blockchain-based services by improving their legal systems and developing public–private cooperation services. In particular, the government is leading the adoption of blockchain in the public sector based on each industry’s usage and secures trust at the national level. By securing blockchain source technology and forming consortiums, major global ICT companies are also commercializing blockchain application services in various fields.

Globally, blockchain can be viewed as a situation in which various industries and fields are convergent and progress past the proof-of-concept stage to the service development and actual commercialization stages. However, despite the emergence of various blockchain technologies and services, their development is slow and their widespread adoption is difficult. Thus, areas with high adoption utility must be identified when using blockchain services in real industries; they must also be applied to similar projects first to create successful cases. Therefore, we must understand the industrial and functional characteristics of blockchain services that can be effectively used and derive directions.

When BCT application services in the public sector are successfully implemented and provided, they can have a significant impact on the overall socioeconomics, and this can simultaneously lead to the vitalization of blockchain applications in the private sector. This study proposes a framework of “service utility evaluation indicators” that can assess the adequacy of the implementation of BCT-based services by considering the characteristics of BCT and platform and their application in the public sector. Based on this, we identify highly effective use cases. This study can promote efficient policies for developing successful blockchain application services by appropriately using blockchain.

The remainder of this paper is structured as follows. The next section presents a literature review regarding the features, benefits, and adoption factors of BCT for the design of the evaluation framework. The subsequent section describes the research methodology that contains three research steps that will be applied. Next, we provide the analysis results, including analytic hierarchy process (AHP) analysis results for weighting the evaluation factors and Delphi survey results. The final section provides our discussion based on the key findings of this study, the theoretical and practical implications, and the paper’s conclusion.

## Background literature

### Blockchain technology

Blockchain is the technology that powers Bitcoin, a popular digital currency built on a peer-to-peer network and cryptographic tools. The Bitcoin network creates a trusting environment in which users can transfer money without relying on central authorities. A blockchain is essentially an append-only data storage of replicated transactions between peers [3]; a distributed transactional database is shared by many parties [4]. Users refer to each other using their public keys to perform transactions and use their private keys to cryptographically sign transactions [5]. Each successful blockchain transaction represents an update to the database, which is replicated and stored by each participant. Transactions are aggregated and appended to the database in blocks, which can be managed automatically using smart contracts [3]. Moreover, decentralized applications (DApps) are services that are based on one or more smart contracts [6]. Bitcoin is a distributed ledger that allows cryptocurrency transactions to be recorded [7]. The distinction between blockchain and other decentralized ledgers is that Bitcoin records new transactions without the intervention of a third party, owing to network participants who collect them and insert them into blocks for verification [8]. Thus, these blocks are linked together by a hash function that allows the entire history of the blocks to be tracked, resulting in a block, which is called a blockchain [9].

The evolution of the blockchain has been a progressive process. Based on their applications, blockchain’s evolution can be divided into Blockchain 1.0, 2.0, and 3.0 [10]. BCT has moved beyond digital currency and into finance and is now being used in health care, supply chain management, market monitoring, smart energy, and copyright protection [11–16]. Blockchain 1.0 is associated with virtual currencies like Bitcoin. Meanwhile, Blockchain 2.0 includes smart contracts, smart property, DApps, decentralized autonomous organizations, and decentralized autonomous corporations (DACs) [7]. Blockchain 3.0 envisions a more advanced form of “smart contracts” to establish a distributed organizational unit that makes and is subject to its laws and operates autonomously [17]. Blockchain 3.0 should be viewed as an evolving and expanding form. This study focuses on the expanded service beyond the 1.0 level to various service areas. In particular, this study examines the appropriate fields and possibilities for applying BCT in the public domain.

### Blockchain benefits and adoption factors

The essential blockchain benefits derived from these functionalities include immutability, non-repudiation, data integrity, transparency, and the potential for equal rights of participants [3, 18–20]. A distributed blockchain application serves as a trusted third party by confirming asset authenticity, authenticating asset ownership, and validating transactions using computer algorithms and cryptography [18, 19]. Blockchains enable organizations to transact directly with each other. Every participating organization has an exact copy of the same digital ledger with a blockchain application. Furthermore, transactions on the shared ledger are immutable, which means that every party can be confident that they are dealing with the same data. With one version of the truth transparently available to all parties, there are no reconciliations, thus enabling faster settlement times and lower transaction costs [6]. As such, blockchains have characteristic benefits and to implement them, stakeholders must understand the factors that influence the adoption of blockchains in organizations [21]. Therefore, to effectively use the advantages of blockchain, the technology–organization–environment (TOE) should be considered together [22].

Table 1 summarizes the blockchain value factors—benefits of BCT and adoption factors that effectively introduce blockchain in organizations and affect the successful development of blockchain application services for the organizations.

**Table 1:** Value and adoption factors of blockchain.

Factor	Characteristic factors	Sources
Values Factors	Auditability, compliance, standardization, process automation, reconciliation, track and trace, data security, data sharing, transparency, trust, resiliency, authenticity, identity management, marketplace creation, new/enhanced products and services, new/expanded partnership, autonomy, scalability, P2P communication, decentralization, observability, easy verification of transactions, comprehensibility of the transactions, data accuracy and reliability, and interoperability	[5, 7, 18–20]
Adoption Factors	Process criticality (associated risk perception), data confidentiality, process reengineering needs, existing technology investments, current data standards (using a common data standard to share information), learning culture, top management support, customer readiness, competitive pressure, government policies, relative advantages, complexity, cost, market dynamics, regulatory support, infrastructural facility, cycle time reduction potential, effort elimination potential (low transaction friction), ancillary benefits, easy verification of transactions, data accuracy, reliability, and new type of inter-organizational collaboration	[5, 18, 19, 21–27]

Meanwhile, existing studies related to blockchain applications generally suggest a decision path based on a series of questions [4, 28]. Recently, several studies [22, 26, 29, 30] have explored blockchain adoption based on the TOE framework. However, in many cases, previous studies have remained only conceptual; they have tested the model for only a specific area case such as supply chain management [4, 9]. Therefore, research on a broader and empirical understanding of the factors that affect the usage of blockchains considering their application in various services is scant. In particular, there is a scarcity of research on blockchain application services in the public sector. Empirical studies in various fields should be conducted before the diffusion and improvement of BCT.

Thus, this study investigates additional useful fields and services based on the characteristics and values of blockchains, and conducts empirical research focusing on cases in the Korean public sector.

## Research methodology

This study develops an evaluation framework for assessing the utility of blockchain application services and verifies the usefulness of the proposed framework by applying it to real use cases. To achieve this, this study investigates the evaluation factors derived from a literature review of blockchain-related studies, proposes a new framework comprising a blockchain service utility evaluation index, and empirically applies them to several use cases. We conducted three research steps in detail: 1) examine the key values of BCT through existing literature and develop a framework of blockchain service utility evaluation index using the AHP method; 2) investigate and select representative blockchain use cases; and 3) apply the evaluation index to use cases and discover highly effective blockchain application service cases using the Delphi method.

This study was approved by the Research Review Board (RRB) of SPRi, which is the research funding institute of this study, prior to initiation. In the review process of the RRB, the research methods (expert FGI, AHP, and Delphi) of this study were clarified in detail and approved without any special issues related to human participation (including the ethical part). In addition, the written informed consent was obtained from all experts involved in FGI, AHP, and Delphi techniques in this study.

### Development of a framework of blockchain service utility evaluation index

To develop a framework for a comprehensive evaluation index, we first researched and referred to the existing literature on specific blockchain value and adoption factors (see Table 1). This study considers evaluation factors for blockchain application services in the public sector, and thus, we reviewed previous research [31] on the level of the socioeconomic impact associated with blockchain adoption.

Subsequently, we discuss the key elements for evaluating the blockchain-based service utility with research, academic, and industry experts who have recently completed blockchain-related project tasks. Table 2 presents the information of five experts who participated. The process of developing the framework through discussions with experts is described as follows. In the first step, a preliminary study was conducted to construct a draft evaluation framework. In the preliminary work, expert advice was provided on the draft evaluation index prepared through literature reviews. The draft of the blockchain service utility evaluation index was verified through an expert advisory meeting. In this process, the framework was reviewed as a whole, and the need for integration between detailed indicators and improvement of the name or definition of the indicator was reviewed. Also, it was considered to add specific explanations and examples for each indicator to further improve the understanding of the indicators. In addition, to construct an effective evaluation framework, it was reviewed to limit the number of detailed evaluation indicators for each subcategory to three representative items. In the second step, it was written in the form of specific key questions for each evaluation index by reflecting the review opinions from the pre-work stage. The final framework was derived through the secondary review process with experts for the complete framework including these key question items. Through this process, a framework constituting “blockchain specialized evaluation index” and “adoption environment evaluation index” was developed (Table 3).

**Table 2:** Information about the expert advisory group.

No	Advisory Division	Job Title	Sector
1	Technology	Senior Researcher	Research
2		Manager	Industry
3	Evaluation Model	Senior Researcher	Research
4		Professor	Academia
5	Business Model	Professor	Academia

**Table 3:** The proposed framework of the blockchain service utility evaluation index.

Category	Subcategory	Evaluation index	Evaluation criteria (key questions)
Blockchain specialized evaluation index	Necessity of use	Need for decentralization	• Is the efficiency (or effectiveness) of the service better when provided in a decentralized form?
		Degree of cooperation	• Is there more added value than the existing service configuration so that various players connecting to this service can participate as network nodes and share the same information?
		Demand for smart contract use	• Is it an appropriate configuration for the service to be supported by a computer algorithm in an automated form instead of a traditional trusted third party or intermediary?
	Ease of application	Oracle risk management	• Are there any alternatives to solve the Oracle risks when ensuring data integrity*? (e.g., Avoidance/Transfer/Reduction/Retention) * Specific data levels for which integrity is required
		Digitalization level	• Are the value chain* information and transaction records used in the service sufficiently digitalized (including the company and its partners)? * Entire service and value chain level
		QoS (Quality of service)	• Does the blockchain platform satisfy the QoS required by the service? • Are the main performance requirements of the service satisfied by the corresponding blockchain platform? (e.g., TPS (transactions per second), execution time, and consensus algorithm)
Adoption environment evaluation index	Acceptability	Maturity of the core technology	• What is the standardization level of core technology and level of technological maturity (including development potential, substitutability, and reliability)?
		The institutional and legal system acceptance level	• Does the government/local government provide legal, institutional, and policy support for the service?
		User acceptance level	• How much will users resist innovation to accept the service? (for checking the users’ positive and negative perceptions of the service)
	Socioeconomic impact	Creation of new markets	• How likely are new markets to be established and created through this service?
		Contribution to industrial development	• How much does such service contribute to the expansion of existing industries?
		Increase in social utility	• How much have the social costs been reduced using this service? (e.g., reduction of history management and supervision costs through traceability and reduction of cost of damage caused by illegal transactions through activation of energy trading platform)

The blockchain specialized evaluation index consists of two subcategories: the necessity of use and the ease of application. The necessity of use is composed of parts that determine the level at which service requires BCT, including decentralization, the degree of cooperation in the value chain, and the demand for smart contracts. The ease of application is summarized as parts that check whether implementing service using the current level of BCT is easy. To confirm these parts, we included Oracle issues, asset digitization level, and quality of service (QoS). The Oracle issue is an indicator to determine whether problems (e.g., data integrity and security problems) that may occur when bringing off-chain data to on-chain can be resolved or avoided.

The adoption environment evaluation index for evaluating the utility of the overall project consists of two subcategories: acceptability and socioeconomic impact. Acceptability encompasses core technology maturity, institutional and legal system acceptance, and user acceptance. The socioeconomic impact is made up of components that are summarized as the creation of new markets, the contribution to industrial development, and the increase in social utility regarding public policy projects.

Finally, we used AHP analysis to determine the priorities and weights for the evaluation factors in Table 2. Saaty’s (1980) AHP is probably the most widely used multicriteria methodology, with numerous applications in diverse fields [32, 33]. When there are multiple evaluation factors, AHP is a hierarchical analysis method that prioritizes multicriteria [34] and is widely used in information system research [34], with AHP-stratified factors considered in decision making and derive relative weights (importance) by pairwise comparison of the evaluation factors for each layer [35, 36]. AHP method is based on comparison matrices resulting from specialists’ evaluations when comparing criteria (alternatives) in pairs. This evaluation requires a certain level of matrix consistency. To assess consistency, the Consistency Ratio (CR) has been used as a measure in many studies [32–38]. To confirm the consistency, Satty [32] proposed a 10% threshold for CR, that is, the recommended value of the CR should be less than 0.1 [32, 38]. As a result of the analysis of this study, all CR values were less than 0.1; the analysis results of these CR values are summarized in Table 6. In this study, the AHP panel consisted of an expert group who had conducted academic research on a blockchain or carried out projects related to blockchain; as shown in Table 4, this expert group was formed by selecting 11 experts in considering specialized fields such as technology/business project evaluation/policy/business modeling. The results of the AHP analysis are presented in the section of analysis results.

**Table 4:** The 11 expert panelists.

No	Advisory Division	Job Title	Sector
1	Technology	General Manager	Industry
2		Senior Researcher	Public Agency
3	Evaluation Model	Doctor	Research
4		Professor	Academia
5		Doctor	Research
6	Policy/Regulation	Lawyer	Industry
7		Director	Public Agency
8		Researcher	Research
9	Business Model	Professor	Academia
10		Director	Research
11		Researcher	Research

### Selection of use cases for validating the framework

**Fig 1:**
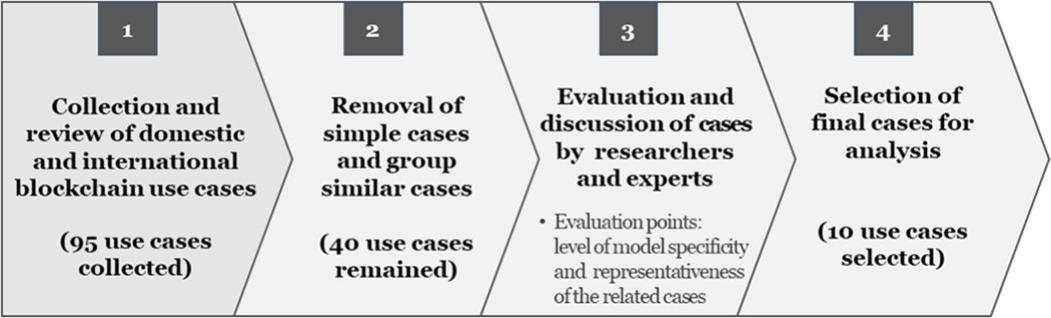
The procedure of selection of blockchain-applied use cases.

We investigated and detailed use cases to demonstrate the effectiveness of our proposed framework. In total, 95 case data were collected through searching domestic and international use case studies. Then, among the 95 cases, 10 representatives were selected to verify the evaluation framework (Fig 1).

The following is a detailed description of the procedure. First, we investigated cases mentioned in domestic and global reports and news articles for use case listing over three months. In Korea, a blockchain pilot project was launched in the public sector in 2017 under the government leadership, so we focused on it and tried to include application cases in various possible fields. In the list-up process, some cases in the private sector were included when they can be applied to the public sector in the future. The use cases were classified by the application field of the service to which the blockchain was applied, and similar service cases in the same field were excluded because the contents were similar, totaling of 95 cases. Then, each case’s one-time or promotional contents were excluded because only the simple service name was presented or the contents were very brief. Of the 95 cases, 40 were identified through this process. For these use cases, the expert panel group and our research team evaluated whether the cases were appropriate for verification, and ultimately, 10 were selected. The level of specificity of the service model and the degree of representativeness of the related cases are the criteria for evaluating and selecting the final use case. Each of the 10 use cases was thoroughly explained, including a service overview, detailed model process, business model (as-is and to-be), the expected effect, and related issues, to allow for expert evaluation. Two detailed use case examples are attached in the (S1 Appendix) for reference. Table 5 summarizes the 10 final use cases.

**Table 5:** The 10 final selected use cases.

	Service Model	Field	Service Overview	Detail Type
1	Blockchain-based peer-to-peer (p2p) Electricity Trading Service Platform	Utilities	A trading platform that supports the distribution of P2P energy by prosumer groups (especially local vendors), including certification of renewable energy supplies and tokenization trading support for carbon credits	p2p Transaction
2	Blockchain-based Shard Car/Parking Lot Platform	Automotive	A platform to support reliable p2p carpool matching based on driver history sharing to increase area-based service and reduce user burden (considering linking/expanding car ownership/license certification, insurance records, and vehicle lifecycle management)	p2p Transaction
3	Blockchain-based Education Service	Education	A service to manage the history of learning information (especially MOOC and blended learning) and test scores to certify each performance and learning module (increase usability by linking instructor history)	Certification
4	Issuance of Notarization for Diplomatic Missions Abroad based on Blockchains	Diplomatic	A service for storing public document information and certificates in the blockchain and sharing them in electronic document form between “Domestic institutions (e.g., banks)—Ministry of Foreign Affairs—Diplomatic missions abroad—Foreign countries”	Certification
5	Blockchain Cloud-based Real Estate Administration Intelligence System	Real estate	A system that when applying for real estate-related loans, bank managers can check real estate information stored in the Korea Financial Telecommunications & Clearings Institute’s blockchain without submitting the relevant certificate to the bank	Deal/Contract
6	Blockchain-based Copyright Protection Service	Property	A service that provides transparent settlement to copyright holders and strengthens content security to create a foundation for legal distribution of works	Copyright deal
7	Blockchain-based Gift Certificate Transaction System	Finance	A blockchain-based mobile gift certificate system that is easy to use and highly secure, and the development of local gift certificates and/or the local currency that reflect the needs of each local government to promote local consumption	Financial transaction
8	Blockchain-based Electronic Voting Service	Voting	A service that stakeholders, such as candidates and observers, can directly verify the voting process and results for enabling reliable online voting	Voting/Certification
9	Blockchain-applied Customs Clearance System	Customs	A system to support real-time import declaration and prevent illegal/tax evasion declaration by improving the declaration and verification process of imported goods	History management
10	Blockchain-based Electric Vehicle Battery Distribution History Management Service	Automotive	A service to support smooth battery recycling and eradication of illegal battery distribution by using of the history management system of electric vehicle batteries used by the (local) government and vendors together	History management/Transactions

This study intends to apply the proposed blockchain service evaluation index framework to real-world use cases to identify highly effective services that can appropriately implement BCT characteristics. A Delphi survey was conducted for this purpose.

### Delphi survey

The Delphi technique is used to predict, diagnose, and solve problems by systematically integrating the opinions of experts on a specific topic [39]. In particular, it is performed when knowledge is incomplete or agreement on a particular topic is lacking [40, 41]. Delphi is not a procedure intended to challenge statistical or model-based procedures. Rather, it is intended for use in judgment and forecasting situations in which pure model-based statistical methods are impractical or feasible due to the lack of adequate historical/economic/technical data, and thus where some form of human judgmental input is necessary [39].

In the Delphi technique, the primary questionnaire mainly uses an unstructured and open-ended response form. This is appropriate when considering the divergent perception of experts and then collecting opinions from it, since the primary purpose of the first questionnaire is deemed the exploration stage. Meanwhile, the modified Delphi technique, in which researchers begin with a structured questionnaire, has also been developed [42]. The pre-structured questionnaire was used in the first Delphi survey in this study, which has the advantage of increasing survey convenience and efficiency even with few surveys. The current study identifies highly effective services that can implement the characteristics of BCT well by applying the blockchain service evaluation index to actual cases. Because many cases needed to be reviewed and evaluated, the first survey used a structured questionnaire. According to previous research, the third questionnaire is considered appropriate because measurement accuracy increases up to the third and then decreases after the fourth [41, 43]. As a result, three rounds of Delphi surveys were conducted in this study.

Delphi studies are usually conducted to achieve a level of agreement among experts about a topic. Using an expert Delphi, this study demonstrates the effectiveness of services organized in use cases. In the process of expert Delphi, experts are asked about their opinions through a standardized questionnaire in many successive rounds [44]. To apply the Delphi technique in this study, a group of experts with in-depth knowledge about blockchain application services was selected, and the expert panel was made up of individuals with similar expertise, such as those who have conducted academic research on a blockchain or have actual project experience. For consistency, the Delphi survey panel was kept the same as the previous AHP expert panel. Delphi was attended by all 11 panelists from the first to third rounds.

## Analysis results

### AHP analysis result for the weights of evaluation factors

As shown in the AHP analysis result (Table 6), the total weight of the “blockchain specialized evaluation index” was 61.8%, which was higher than that of the “adoption environment evaluation index” (38.2%). In particular, the most important part is “ease of application” (47.5%), which is an aspect of how ready to apply. The remaining indicators were analyzed in the order of “socioeconomic impact” (22.4%), “acceptability” (15.8%), and “necessity of use” (14.3%).

**Table 6:** AHP result for the weights of evaluation factors.

Category	Subcategory	Weight (%)	CR of Tier 1	Evaluation Index	Weight (%)	CR of Tier 2
Blockchain specialized evaluation index	Necessity of use	14.3	0.018	Need for decentralization	38.8	0.003
				Degree of cooperation	48.3	
				Demand for smart contract use	12.9	
	Ease of application	47.5		Oracle risk management	50.0	0.006
				Digitalization level	24.1	
				QoS (Quality of service)	25.9	
Adoption environment evaluation index	Acceptability	15.8		Maturity of the core technology	52.3	0.015
				The institutional and legal system acceptance level	31.7	
				User acceptance level	16.1	
	Socioeconomic impact	22.4		Creation of new markets	47.0	0.002
				Contribution to industrial development	27.9	
				Increase in social utility	25.1	

In the analysis results for each subcategory, within “necessity of use,” the “degree of cooperation,” which is an aspect of information sharing, occupied the largest portion with 48.3%. Next, “necessity for decentralization” followed with 38.8%. This result shows that the core of blockchain application is a fair interaction with more stakeholders than the existing centralized method. This result also indicates that open innovation can be more active in blockchain applications. In the subcategory of “ease of application,” Oracle risk management had the highest weight at 50%. Due to BCT, such as reliability, immutability, and trust, this result is interpreted as emphasizing that if the stored information is incorrect, it can become a serious problem.

Meanwhile, in the subcategory of “acceptability,” the “maturity of core technology” (52.3%) was evaluated as the highest, followed by “institutional and legal system acceptance level” (31.7%) and “user acceptance level” (16.1%). This indicates that the blockchain still should enhance the maturity of technology, which requires verification. This can also be interpreted as an important factor not only whether BCT with the characteristics of infrastructure can be applied and used but also whether it is built based on institutional and legal systems. In the subcategory of “socioeconomic impact,” the “creation of new markets” was the highest at 47.0%. This shows that blockchain-related product and service markets can be generated as an important factor. In our analysis results, all CR values were less than 0.1, which is the recommended standard [32, 38]. Therefore, the consistency evaluation criteria were satisfied.

### Delphi survey results

In this study, we used the Delphi technique to apply our proposed framework to 10 actual use cases and identified highly effective services that can well implement the characteristics of BCT. A consensus was reached by performing the Delphi over three rounds of the expert panel. Detailed data on the Delphi results from the first to the third round are presented in the (S1 Data). Table 7 displays the results of the Delphi surveys. The values are calculated by adding the weights of the AHP analysis results summarized in Table 6 to the values assigned by the experts for 12 evaluation indicators for each of the four evaluation subcategories per service use case.

**Table 7:** Delphi results of utility evaluation for blockchain application services.

Service model	Score *(weighted for each index)*				Total Score (After weighting)	Rank
	Necessity of use	Ease of application	Acceptability	Socioeconomic impact		
Blockchain-based p2p Electricity Trading Service Platform	24.15	29.71	22.30	28.90	105.06	1
Blockchain-applied Customs Clearance System	24.56	27.80	24.98	25.18	102.52	2
Blockchain-based Electronic Voting Service	25.74	27.30	23.18	22.66	98.88	3
Blockchain Cloud-based Real Estate Administration Intelligence System	23.34	27.55	22.06	24.70	97.65	4
Blockchain-based Gift Certificate Transaction System	21.93	27.30	22.30	25.66	97.19	5
Blockchain-based Copyright Protection Service	21.17	24.78	22.08	26.02	94.05	6
Blockchain-based Shard Car/Parking Lot Platform	22.87	25.44	21.21	23.67	93.20	7
Issuance of Notarization for Diplomatic Missions Abroad based on Blockchains	21.28	23.60	20.30	16.22	81.40	8
Blockchain-based Electric Vehicle Battery Distribution History Management Service	19.76	21.70	20.30	19.58	81.34	9
Blockchain-based Education Service	15.41	23.60	18.53	18.74	76.28	10

Results of blockchain service utility evaluation for the use cases in Table 7 reveal that services such as “blockchain-based p2p electricity trading service platform,” “blockchain-applied customs clearance system,” and “blockchain-based electronic voting service” have high utility. Most of the top five services, in particular, had relatively high scores in the evaluation index subcategories of “ease of application,” “necessity of use,” and “socioeconomic impact.” Additionally, as a result of comparing the difference in the average between the service group in the top 5 (group H) and top 6 or lower (group L), in the case of the average value of “ease of application,” group H was 4.1 points higher than group L, for “socioeconomic impact,” group H had an average value higher than group L by 4.6 points, and for “necessity of use,” the average value of group H was 3.8 points higher than that of group L. These evaluation results show that services with a high utility score have a high likelihood of business feasibility and added value creation using blockchain regarding less complex applications, business needs, and impact. The best-rated “p2p electricity trading service platform” is a type of p2p transaction, which is a typical and valuable feature of blockchain to use in business. Additionally, the Delphi result can predict that the utility would be high for services where reliability is an important element, such as customs clearance system, electronic voting, copyright protection, real estate administration, and gift certificate.

Meanwhile, examining the degree of change in the overall ranking by the weight of the evaluation index yielded some fluctuations in ranking when “ease of application” was high. Tables 3 and 6 summarize that “ease of application” is linked to Oracle issues, asset digitalization level, and QoS. This shows that it is an important criterion for judging whether the technology or environment required for the service is properly ready.

Since BCT is not yet fully mature or fully prepared for developing new services, the applicability and acceptability of the blockchain to the current situation in the relevant business must be considered. Therefore, when the scores for ease of application and acceptability are high, a certain level of the base environment for BCT adoption is expected to be established.

## Discussion

As the blockchain-related market expands, domestic and international interests shift from a simple application to commercialization, and expectations for practical effects increase. Accordingly, a more specific response to “why blockchain should be used” is required. This study investigated the value of blockchain application services as enablers that can create new utilities based on blockchain characteristics. Our results confirmed that the need for decentralization, cooperation model level, the degree of the Oracle problem, and the digitization of assets should be considered. Fundamentally, blockchain can be more useful when it is based on improving existing processes. Both at home and abroad, people believe that blockchain is a young technology. Therefore, for this technology to be properly applied, a blockchain-friendly business environment must be supported. The evaluation indicators presented as the ease of application in this study are related to the levels of maturity and preparation of technology and the market. The reason why experts rated the ease of application as the most important in this study can be interpreted in the same way.

This research predicts the utility of blockchain-based services and first applies BCT to services that are more appropriate. We have organized evaluation factors by category for this purpose, so that blockchain application services can be investigated comprehensively rather than in the form of a fragmentary decision tree of previous studies. For example, Pedersen et al. [4] suggested a decision tree framework that determines whether the application of BCT is justified. On the decision tree, each question item is answered with a binary “Yes” or “No.” If the answer to any item is “No,” it tends to be decided simply with the conclusion that BCT is not required. Such a fragmented decision path-based framework may not provide a comprehensive review of various factors that should be considered for whether BCT is applied effectively and efficiently to the services. In addition, this existing research has only studied how the framework is used to develop a blockchain prototype for the special area of the maritime shipping industry, is a conceptual study, and has not been empirically verified. Meanwhile, some studies on BCT adoption have used the TOE framework, for example, Kulkarni et al. [22] examined the factors affecting the adoption of BCT in banking services based on the TOE framework. The study collected data from employees of banks and the financial services industry and verified it with a quantitative methodology, showing that technological, organizational, and environmental factors can influence BCT adoption. However, this existing study was limited to banking and financial services. Accordingly, there is a limit to deriving more comprehensive factors that can be applied to various areas. As such, there are some existing studies on factors related to BCT adoption and application, but these studies were limited to developing a conceptual framework and not empirically verified or limited to only a specific area.

This study differentiates from previous studies in how it systematically presents a framework from a comprehensive perspective that considers the specialized aspects of blockchain, the suitability for blockchain adoption, and potential ripple effects based on the characteristics of a new technology of blockchain. In this study, the evaluation framework was structured in two dimensions (blockchain specialized and adoption environment) and four perspectives (necessity of use, ease of application, acceptability, and socioeconomic impact) by organizing the features that appear in various forms in the literature. It also presented 12 indicators that can be measured for each. The indicators can be differentiated from existing studies in that they provide a basis for the theory to be proven and to be connected to actual implementation cases. While most of the existing studies have been conducted in the form of presenting concepts or conducting case studies on a specific field, this study contributes theoretically by presenting an overarching viewpoint related to the use of BCT in various areas, and by presenting a framework that can systematically examine the utility of blockchain application services and has been empirically verified through application to actual use cases.

This study also provides practical implications. The proposed framework presents practical value as a useful reference for decision-makers considering blockchain adoption in various service models. In particular, when it is necessary to preemptively promote policies to support industrial development, various perspectives besides profitability, such as return on investment, should be investigated, and this study’s framework provides such. This study’s findings showed that the necessity of use and ease of application (regarding suitability for adoption) can be mutually complementary indicators as necessary or sufficient conditions for applying blockchain to services. As a result, blockchain service providers must first determine whether this requirement is met by applying the “necessity to use the specialized features of the blockchain” indicator to the business that they wish to implement. As suggested in this study, stakeholders must determine whether service requires decentralization and collaboration with departments, institutions, and companies involved in the value chain. Moreover, whether the requirements for data integrity or reliability enhancement are met during this process must also be examined. Subsequently, they must determine whether the service or business environment is properly configured or can be supplemented based on the indicator of ease of application. This study suggests considering whether the environment requires more technological maturity. With the Fourth Industrial Revolution on a full-fledged trajectory, the digital transformation of industries and the economy is rapidly spreading. However, because the speed of digital transformation varies depending on the industry or service area, including the situation of domestic and global companies, a more sophisticated review of each business model is required. Moreover, in such a period, policy efforts to promote industries from a mid- to long-term perspective by emphasizing the intrinsic value and potential of new technology are required.

**Fig 2:**
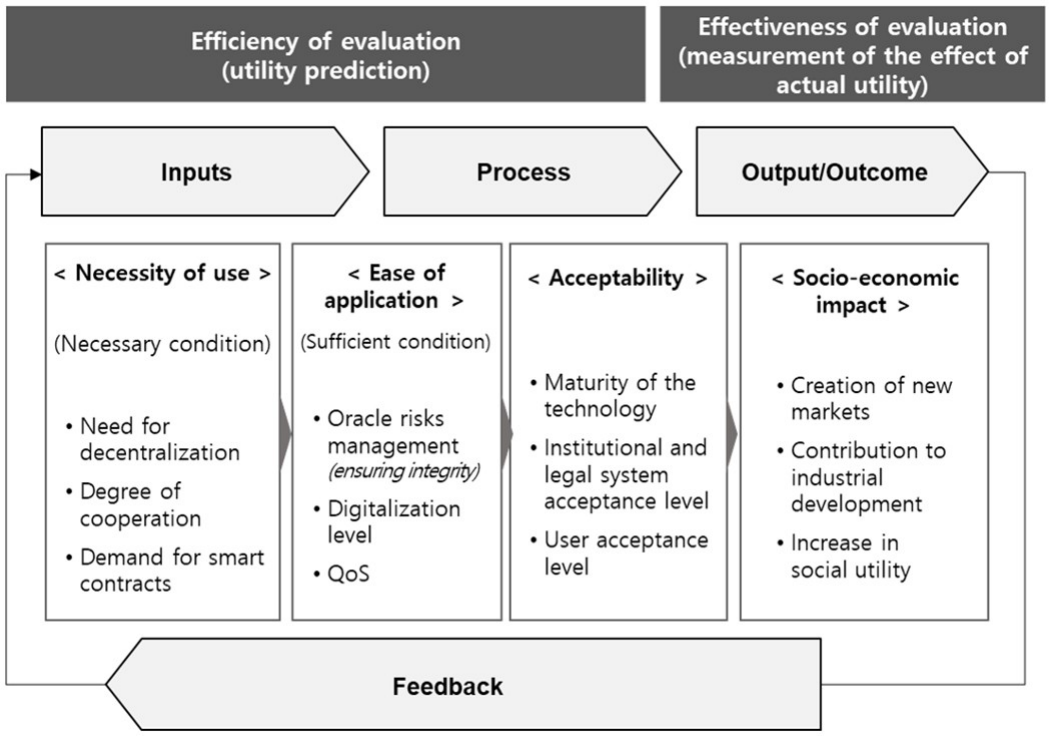
Utility evaluation factors with IPO logic model.

The evaluation indicators derived through this study can be substituted for the procedure of adopting and reviewing the effects of blockchain services. Concerning public sector project evaluation, it is generally based on the IPO logic model of Input → Process → Output/Outcome (see Fig 2). The model for utility review can become clearer if the items of the proposed evaluation index are applied to the IPO model. In this study, “necessity of use” corresponds to input, and “ease of application (suitability for adoption)” can be viewed as input and process components. The term “inputs” refers to the organization’s controllable resources and assets, and actual inputs may include human resources, physical assets, and so on. We are currently focusing on technical characteristics, but as the project progresses, we can add human and material resources capable of properly adopting the technology. In organizations, “process” refers to optimizing the function as a whole, rather than ancillary optimization of detailed processes. Meanwhile, the acceptability suggested, such as core technology standardization, institutional/legislative acceptance, and user acceptance, can serve as the foundation for such process optimization. Moreover, the “Output and Outcome” aspects are reflected as indicators of the socioeconomic impact of this study. For general public projects, Output measures the direct effects of individual projects (e.g., number of executions and supports, and number of participants), whereas Outcome includes benefits and secondary effects (e.g., production inducement and job creation). This study proposed socioeconomic impact indicators that include more comprehensive Output and Outcome. Accordingly, by applying the utility evaluation index factors of this study to the IPO logic model (Fig 2), more systematic management of developing services can be established to evaluate the adequacy of the implementation of BCT-based services in the public sector.

## Conclusion

While BCT is attracting attention because it is used to improve existing industry processes and create innovative new services, this study investigated the factors that should be considered when applying the business by considering the characteristics of the BCT at this point. In this study, the factors of the framework of blockchain service utility evaluation indexes were derived through an existing literature review and expert discussion process, and the evaluation indicators were verified and weighted according to the assessment by the expert panel through the AHP. Additionally, the applicability of the proposed evaluation framework was verified by applying evaluation indicators to use cases through the expert Delphi method.

However, this study has some limitations. The indicators derived in this study consist of mainly qualitative aspects, which were considered and evaluated based on the insights of experts who have experienced blockchain businesses when evaluating use cases. If quantitative components can be converted or added to the current indicators, a more objective evaluation will be possible in the future. This research was conducted in a single country. However, the analysis results of the proposed framework may differ depending on the country’s policy and the market’s maturity. Thus, conducting a comparative study across multiple countries in future research may provide more interesting insights. Additionally, the follow-up studies may consider the degree of technical governance for the platform and business governance, including domain requirements for each business and service in countries.

There are various attempts to apply blockchain to industries. Korea has also conducted various pilot projects through public-leading blockchain projects that focus on “creating an ecosystem base” by securing best practices and reorganizing regulations for each industry. In the future, we believe that we should focus on the direction of securing mid- to long-term competitiveness in the industry by linking this momentum to the paradigm of digital transformation. For the core business model discovered through the blockchain pilot project to be extended to the commercialization stage, a continuous support and evaluation system is needed. Additionally, for the support of each stage of corporate growth, stakeholders must establish a support and evaluation system for the entire ecosystem. Although blockchain is rapidly evolving, it is important to remember that it is still a nascent market. Concerning convergence with major core technologies, leading through inter-ministerial cooperation in the public sector, while reviewing BCT expansion is still necessary. This study’s evaluation framework focuses on applying BCT to existing industries to generate more valuable business. This approach will allow new technologies to hit the ground running in the existing economy more quickly. The balanced view on the necessity for the value of BCT and the readiness of the domain to be applied, presented in this study, is expected to contribute to the development of competitive blockchain application services in both the public sector and the entire industry.

## Supporting information

S1 Appendix Detailed use case examples. (PDF)

S1 Data Delphi expert survey data. (PDF)
