# Supplementary material for: Priority evaluation factors for blockchain application services in public sectors
Source: PLoS One. 2023 Mar 2;18(3):e0279445. doi: 10.1371/journal.pone.0279445 (PMC9980796; doi:10.1371/journal.pone.0279445)
Supplement: S1 Appendix — (PDF) [file pone.0279445.s001.pdf]

**S1 Appendix. Detailed use case examples**

<table>
<tr><th colspan="2">Use case Name</th><th>Blockchain-applied Customs Clearance System</th></tr>
<tr><td rowspan="3">Overview</td><td>Service Concept Overview</td><td>• Service in the public sector where participating organizations create and share data related to import and export logistics using blockchain technology from the export/import contract stage to delivery</td></tr>
<tr><td>Background</td><td>• As the number of e-commerce imports is rapidly increasing, shipping companies must manage and submit a list of customs information for every 28 types of goods, which is a combination of the order information of the e-commerce companies and their transportation information and submit it at the customs office; however, most of the work is done manually, which is a problem</td></tr>
<tr><td>Necessity</td><td>• Regarding the delivery of product information between transport and e-commerce companies and the creation of inventory customs clearance by transport companies, there is a constant possibility of low-priced and false declarations</td></tr>
<tr><td>The system process</td><td>The detailed process at each step</td><td>• Providing blockchain-based import/export customs logistics service with the participation of companies and institutions related to import and export, such as shippers, shipping companies, terminals, customs companies, forwarders, transport companies, and insurance companies<br>- (Step 1) Real-time sharing of customs-related data owned by e-commerce companies and express delivery companies to the National Customs Service through blockchain<br>- (Step 2) E-commerce companies and express delivery companies share customs-related data with the National Customs Service and participants through the blockchain reporting portal with the ID they received from the National Customs Service and check the progress of the report<br>- (Step 3) By automatically generating import declaration data by combining the information of the goods that the e-commerce company has requested to be delivered to the express company and the shipment information of the goods shipped by the express company, the data is received and processed through Uni-Pass and customs clearance</td></tr>
<tr><td rowspan="3">Business model</td><td colspan="2">• Establishment and usage of customs clearance system with essential application of blockchain-based forgery technology</td></tr>
<tr><td>As-Is</td><td>• All reporting companies make handwritten documents based on information provided by e-commerce companies, so they are vulnerable to forgery or falsification</td></tr>
<tr><td>To-Be</td><td>• The contents of the declaration form are sequentially filled based on the production information of each participant, and a customs clearance system that cannot be forged, or altered by the will of a specific person, is established</td></tr>
</table>

| | |
|---|---|
| **Expected effect** | • Increase in the transparency and efficiency of the trade procedure to secure trade stability, real-time edibility, and simplification of business procedures<br>- Smart contract-based business automation and prevention of trade fraud caused by malicious document manipulation are expected<br>- It is expected that a significant portion of the customs and customs duties will be converted to blockchain-based |
| **Related issue** | • Changes in customs procedures according to cases reported directly by individuals, setting the scope of information to be provided, and seeking ways to cooperate with companies subject to special customs clearance and express delivery companies |

<table>
<tr><th colspan="2">Use case Name</th><th>Blockchain-based Electric Vehicle Battery Distribution History Management Service</th></tr>
<tr><td rowspan="3">Overview</td><td>Service Concept Overview</td><td>• Service in the public sector to eradicate illegal battery distribution and illegal tuning through a blockchain history system and standardization of waste batteries</td></tr>
<tr><td>Background</td><td>• Depending on the usage environment context, the performance of the battery will decrease compared to the initial stage, and when the performance decreases by 20–30%, it is defined as the end of the battery life; it can be recycled for ESS (energy storage system)</td></tr>
<tr><td>Necessity</td><td>• Due to the spread of electric vehicles and their life cycle, the number of electric vehicle waste batteries is expected to increase in the future, but there is no guarantee of safety in recycling and distribution<br>- It is necessary to ensure the reliability and safety of waste battery history information, such as recovery-classification-grading and reusable amount information in the recycling and distribution of waste batteries for electric vehicles<br>- It is necessary to provide historical information on waste batteries through ESS or built-in module-specific QR codes and to check history information transparently</td></tr>
<tr><td>The system process</td><td>The detailed process at each step</td><td>• By using the necessary modules for each service target, a blockchain service that provides transaction creation and inquiry functions is built, and the level of asset digitization is high and free from Oracle issues by embedding QR codes for each module<br>- (Step 1) When an electric vehicle user returns a waste battery and is placed in the waste battery center, the battery is inspected and analyzed for each vehicle type, the residual value is determined, and then the pack module is disassembled<br>➢ If vehicle information is entered through the DApp when used electric vehicle batteries are received, a transaction is created that records the existing registered company information, the newly created pack number, and the current time</td></tr>
</table>

| | | |
|---|---|---|
| | | - (Step 2) After grading according to the pack/module analysis results, stability tests are performed through destructive and non-destructive tests<br>➢ When analyzing waste battery center packs and modules, analysis information (SOH, SOC, SOP, SOB) is input through the DApp to create pack number and time record transactions<br>- (Step 3) Battery safety packaging is processed according to the results of classification, safety inspection, and application system for each grade and ESS reuse, and defective batteries are discarded |
| **Business model** | • Through the blockchain-based EV battery distribution history service, it is possible to accurately predict the usage period of the waste battery and reuse it as a home/industrial ESS, which is expected to reduce costs and prevent environmental problems | |
| | **As-Is** | • Currently, there is a problem with their use of batteries as the generation of waste batteries for electric vehicles increases due to the spread of electric vehicles |
| | **To-Be** | • It is expected that a system will be established to manage the overall history of disposal, management, and distribution of waste batteries after the use of electric vehicles, thereby enhancing the stability of the distribution of waste batteries, reducing costs, and creating added values such as environmental protection |
| **Expected effect** | | • When distributing ESS from recycled waste batteries for electric vehicles, it is possible to reduce costs and expand the introduction of electric vehicles in the long term by ensuring distribution safety and transparency<br>- Contributing to the reduction of ESS introduction due to dissemination through the reuse of waste batteries and contributing to the vitalization of the ESS industry and the recycled battery industry<br>- Through the expansion and strengthening of the demand market for reusable batteries, domestic companies can expand their exports and invest as resource circulation is improved and cost access is lowered<br>- Realization of a leading ICT national image by successfully establishing a process model for manufacturing waste batteries following the spread of the global trend of the electric vehicle market |
| **Related issue** | | • It is important to consider social issues regarding legal, regulatory, and institutional issues; legal acceptance; and social and user acceptance<br>- As it is not a service model that deals with personal information, there are no conflicting elements and security issues with the “Personal Information Protection Act,” so legal acceptance is relatively high |
