# Supplementary material for: Priority evaluation factors for blockchain application services in public sectors
Source: PLoS One. 2023 Mar 2;18(3):e0279445. doi: 10.1371/journal.pone.0279445 (PMC9980796; doi:10.1371/journal.pone.0279445)
Supplement: S1 Data — (PDF) [file pone.0279445.s002.pdf]

S1 Data. Delphi expert survey data

1) Delphi 1st round

Service Model	Necessity of use *(out of 21 points)*	Ease of application *(out of 21 points)*	Acceptability *(out of 21 points)*	Socio-economic impact *(out of 21 points)*	Sum
Blockchain-applied Customs Clearance System	15.65	14.01	15.55	15.28	60.49
Blockchain-based Electric Vehicle Battery Distribution History Management Service	11.65	11.20	13.11	12.38	48.34
Blockchain Cloud-based Real Estate Administration Intelligence System	15.20	13.92	14.84	15.29	59.25
Issuance of Notarization for Diplomatic Missions Abroad based on Blockchains	13.19	12.56	13.66	10.75	50.16
Blockchain-based peer-to-peer (p2p) Electricity Trading Service Platform	15.47	15.11	14.10	14.64	59.32
Blockchain-based Electronic Voting Service	17.38	13.91	14.83	14.30	60.42
Blockchain-based Copyright Protection Service	13.66	13.01	14.01	16.02	56.70
Blockchain-based Gift Certificate Transaction System	14.93	13.83	14.65	13.47	56.88
Blockchain-based Shard Car/Parking Lot Platform	14.29	12.74	13.28	14.10	54.41
Blockchain-based Education Service	9.65	12.20	11.29	8.37	41.51

2) Delphi 2nd round

Service Model	Necessity of use	Ease of application	Acceptability	Socio-economic impact	Sum
Blockchain-applied Customs Clearance System	16.30	14.11	16.20	15.30	61.91
Blockchain-based Electric Vehicle Battery Distribution History Management Service	13.00	11.10	13.00	12.00	49.10
Blockchain Cloud-based Real Estate Administration Intelligence System	15.20	14.01	14.20	15.10	58.51
Issuance of Notarization for Diplomatic Missions Abroad based on Blockchains	14.00	11.91	13.20	10.10	49.21
Blockchain-based peer-to-peer (p2p) Electricity Trading Service Platform	16.20	15.11	14.39	16.65	62.35
Blockchain-based Electronic Voting Service	17.00	14.01	15.30	14.20	60.51
Blockchain-based Copyright Protection Service	14.00	12.91	14.10	16.00	57.01
Blockchain-based Gift Certificate Transaction System	14.30	14.00	14.30	14.28	56.88
Blockchain-based Shard Car/Parking Lot Platform	15.00	13.00	13.91	13.83	55.74
Blockchain-based Education Service	10.19	12.00	12.00	10.29	44.48

3) Delphi 3rd round

Service Model	Necessity of use	Ease of application	Acceptability	Socio-economic impact	Sum
Blockchain-applied Customs Clearance System	16.00	14.29	16.29	15.42	62.00
Blockchain-based Electric Vehicle Battery Distribution History Management Service	13.145	11.145	13.00	12.00	49.29
Blockchain Cloud-based Real Estate Administration Intelligence System	15.29	14.14	14.29	15.14	58.86
Issuance of Notarization for Diplomatic Missions Abroad based on Blockchains	14.00	12.00	13.00	10.00	49.00
Blockchain-based peer-to-peer (p2p) Electricity Trading Service Platform	16.00	15.14	14.43	17.72	63.29
Blockchain-based Electronic Voting Service	17.00	14.00	15.00	14.00	60.00
Blockchain-based Copyright Protection Service	14.00	12.85	14.29	16.00	57.14
Blockchain-based Gift Certificate Transaction System	14.43	14.00	14.43	15.71	58.57
Blockchain-based Shard Car/Parking Lot Platform	15.00	13.00	13.72	14.57	56.29
Blockchain-based Education Service	10.00	12.00	12.00	11.43	45.43

3-1) Delphi results with weighted score

Service Model	Category	Index	Score (a) *(out of 7 points)*	Sub-Total *(out of 21 points)*	Total	Index Weight (b)	Weighted Score (a+(a*b))	Weighted Sub-Total (c)	Category Weight (d)	Category Score (c+(c*d))	Total Score (Atfer weighting)	Rank (Atfer weighting)
Blockchain -applied Customs Clearance System	(A) Necessity of use	(1) Need for decentralization	5.00	16.00	62.00	38.8%	6.94	21.49	14.3%	24.56	102.52	2
		(2) Degree of cooperation	6.00			48.3%	8.90					
		(3) Demand for smart contracts use	5.00			12.9%	5.65					
	(B) Ease of application	(4) Oracle risks management	4.00	14.29		50.0%	6.00	18.85	47.5%	27.80		
		(5) Digitalization level	5.145			24.1%	6.38					
		(6) QoS	5.145			25.9%	6.47					
	(C) Accept -ability	(7) Maturity of the core technology	5.145	16.29		52.3%	7.83	21.57	15.8%	24.98		
		(8) Institutional and legal system acceptance level	5.145			31.7%	6.77					
		(9) User acceptance level	6.00			16.1%	6.97					
	(D) Socio economic impact	(10) Creation of new markets	5.14	15.42		47.0%	7.56	20.57	22.4%	25.18		
		(11) Contribution to industrial development	5.14			27.9%	6.58					
		(12) Increase in social utility	5.14			25.1%	6.43					

Blockchain -based Electric Vehicle Battery Distribution History Management Service	(A)	(1)	4.00	13.145	49.29	38.8%	5.55	17.29	14.3%	19.76	81.34	9
		(2)	4.00			48.3%	5.93					
		(3)	5.145			12.9%	5.81					
	(B)	(4)	3.145	11.145		50.0%	4.71	14.71	47.5%	21.70		
		(5)	4.00			24.1%	4.96					
		(6)	4.00			25.9%	5.04					
	(C)	(7)	5.00	13.00		52.3%	7.62	17.53	15.8%	20.30		
		(8)	4.00			31.7%	5.27					
		(9)	4.00			16.1%	4.64					
	(D)	(10)	4.00	12.00		47.0%	5.88	16.00	22.4%	19.58		
		(11)	4.00			27.9%	5.12					
		(12)	4.00			25.1%	5.00					
Blockchain Cloud -based Real Estate Admin Intelligence System	(A)	(1)	5.145	15.29	58.86	38.8%	7.14	20.42	14.3%	23.34	97.65	4
		(2)	5.145			48.3%	7.63					
		(3)	5.00			12.9%	5.65					
	(B)	(4)	4.00	14.14		50.0%	6.00	18.68	47.5%	27.55		
		(5)	5.14			24.1%	6.38					
		(6)	5.00			25.9%	6.30					
	(C)	(7)	5.00	14.29		52.3%	7.62	19.05	15.8%	22.06		
		(8)	4.145			31.7%	5.46					
		(9)	5.145			16.1%	5.97					
	(D)	(10)	5.00	15.14		47.0%	7.35	20.18	22.4%	24.70		
		(11)	5.00			27.9%	6.40					
		(12)	5.14			25.1%	6.43					

Issuance of Notarization for Diplomatic Missions Abroad based on Blockchains	(A)	(1)	4.00	14.00	49.00	38.8%	5.55	18.62	14.3%	21.28	81.40	8
		(2)	5.00			48.3%	7.42					
		(3)	5.00			12.9%	5.65					
	(B)	(4)	4.00	12.00		50.0%	6.00	16.00	47.5%	23.60		
		(5)	4.00			24.1%	4.96					
		(6)	4.00			25.9%	5.04					
	(C)	(7)	5.00	13.00		52.3%	7.62	17.53	15.8%	20.30		
		(8)	4.00			31.7%	5.27					
		(9)	4.00			16.1%	4.64					
	(D)	(10)	3.00	13.00		47.0%	4.41	13.25	22.4%	16.22		
		(11)	3.00			27.9%	3.84					
		(12)	4.00			25.1%	5.00					
Blockchain -based p2p Electricity Trading Service Platform	(A)	(1)	5.00	16.00	63.29	38.8%	6.94	21.13	14.3%	24.15	105.06	1
		(2)	5.00			48.3%	7.42					
		(3)	6.00			12.9%	6.77					
	(B)	(4)	4.85	15.14		50.0%	7.29	20.14	47.5%	29.71		
		(5)	5.145			24.1%	6.38					
		(6)	5.145			25.9%	6.47					
	(C)	(7)	5.145	14.43		52.3%	7.83	19.26	15.8%	22.30		
		(8)	4.14			31.7%	5.46					
		(9)	5.145			16.1%	5.97					
	(D)	(10)	5.86	17.72		47.0%	8.61	23.61	22.4%	28.90		
		(11)	6.00			27.9%	7.67					
		(12)	5.86			25.1%	7.33					

Blockchain -based Electronic Voting Service	(A)	(1)	6.00	17.00	60.00	38.8%	8.33	22.52	14.3%	25.74	98.88	3
		(2)	5.00			48.3%	7.42					
		(3)	6.00			12.9%	6.77					
	(B)	(4)	4.00	14.00		50.0%	6.00	18.51	47.5%	27.30		
		(5)	5.00			24.1%	6.21					
		(6)	5.00			25.9%	6.30					
	(C)	(7)	5.00	15.00		52.3%	7.62	20.02	15.8%	23.18		
		(8)	5.00			31.7%	6.59					
		(9)	5.00			16.1%	5.81					
	(D)	(10)	4.00	14.00		47.0%	5.88	18.51	22.4%	22.66		
		(11)	4.00			27.9%	5.12					
		(12)	6.00			25.1%	7.51					
Blockchain -based Copyright Protection Service	(A)	(1)	5.00	14.00	57.14	38.8%	6.94	18.52	14.3%	21.17	94.05	6
		(2)	4.00			48.3%	5.93					
		(3)	5.00			12.9%	5.65					
	(B)	(4)	2.85	12.85		50.0%	4.29	16.80	47.5%	24.78		
		(5)	5.00			24.1%	6.21					
		(6)	5.00			25.9%	6.30					
	(C)	(7)	5.00	14.29		52.3%	7.62	19.07	15.8%	22.08		
		(8)	4.29			31.7%	5.64					
		(9)	5.00			16.1%	5.81					
	(D)	(10)	5.00	16.00		47.0%	7.35	21.26	22.4%	26.02		
		(11)	5.00			27.9%	6.40					
		(12)	6.00			25.1%	7.51					

Blockchain -based Gift Certificate Transaction System	(A)	(1)	4.14	14.43	58.57	38.8%	5.75	19.19	14.3%	21.93	97.19	5
		(2)	5.145			48.3%	7.63					
		(3)	5.145			12.9%	5.81					
	(B)	(4)	4.00	14.00		50.0%	6.00	18.51	47.5%	27.30		
		(5)	5.00			24.1%	6.21					
		(6)	5.00			25.9%	6.30					
	(C)	(7)	5.145	14.43		52.3%	7.83	19.26	15.8%	22.30		
		(8)	4.14			31.7%	5.46					
		(9)	5.145			16.1%	5.97					
	(D)	(10)	5.29	15.71		47.0%	7.77	20.96	22.4%	25.66		
		(11)	5.13			27.9%	6.58					
		(12)	5.29			25.1%	6.61					
Blockchain -based Shard Car /Parking Lot Platform	(A)	(1)	5.00	15.00	56.29	38.8%	6.94	20.01	14.3%	22.87	93.20	7
		(2)	5.00			48.3%	7.42					
		(3)	5.00			12.9%	5.65					
	(B)	(4)	4.00	13.00		50.0%	6.00	17.25	47.5%	25.44		
		(5)	5.00			24.1%	6.21					
		(6)	4.00			25.9%	5.04					
	(C)	(7)	5.00	13.72		52.3%	7.62	18.32	15.8%	21.21		
		(8)	3.72			31.7%	4.89					
		(9)	5.00			16.1%	5.81					
	(D)	(10)	4.43	14.57		47.0%	6.51	19.34	22.4%	23.67		
		(11)	5.00			27.9%	6.40					
		(12)	5.14			25.1%	6.43					

Blockchain-based Education Service	(A)	(1)	3.00	10.00	45.43	38.8%	4.16	13.48	14.3%	15.41	76.28	10
		(2)	4.00			48.3%	5.93					
		(3)	3.00			12.9%	3.39					
	(B)	(4)	4.00	12.00		50.0%	6.00	16.00	47.5%	23.60		
		(5)	4.00			24.1%	4.96					
		(6)	4.00			25.9%	5.04					
	(C)	(7)	4.00	12.00		52.3%	6.09	16.00	15.8%	18.53		
		(8)	4.00			31.7%	5.27					
		(9)	4.00			16.1%	4.64					
	(D)	(10)	4.14	11.43		47.0%	6.09	15.31	22.4%	18.74		
		(11)	3.57			27.9%	4.57					
		(12)	3.72			25.1%	4.65					
